# Participation of the IL-10RB Related Cytokines, IL-22 and IFN-λ in Defense of the Airway Mucosal Barrier

**DOI:** 10.3389/fcimb.2020.00300

**Published:** 2020-06-19

**Authors:** Danielle Ahn, Alice Prince

**Affiliations:** Department of Pediatrics, Columbia University Medical Center, New York, NY, United States

**Keywords:** mucosal barriers, respiratory epithelial barrier, *Staphylococcus aureus*, *Pseudomonas aeruginosa*, *Klebsiella pneumoniae*, influenza, coronavirus, ESKAPE pathogens

## Abstract

The airway epithelial barrier is a major barrier protecting against clinically significant infections of the lung. Its integrity is often compromised due to mechanical, chemical, or infectious causes. Opportunistic bacterial pathogens are poised to cause parenchymal infection and become difficult to eradicate due to adaptive metabolic changes, biofilm formation, and the acquisition of antimicrobial resistance and fitness genes. Enhancing mucosal defenses by modulating the cytokines that regulate barrier functions, such as interleukin-22 (IL-22) and interferon-λ (IFN-λ), members of the IL-10 family of cytokines, is an attractive approach to prevent these infections that are associated with high morbidity and mortality. These cytokines both signal through the cognate receptor IL-10RB, have related protein structures and common downstream signaling suggesting shared roles in host respiratory defense. They are typically co-expressed in multiple models of infections, but with differing kinetics. IL-22 has an important role in the producing antimicrobial peptides, upregulating expression of junctional proteins in the airway epithelium and working in concert with other inflammatory cytokines such as IL-17. Conversely, IFN-λ, a potent antiviral in influenza infection with pro-inflammatory properties, appears to decrease junctional integrity allowing for bacterial and immune cell translocation. The effects of these cytokines are pleotropic, with pathogen and tissue specific consequences. Understanding how these cytokines work in the mucosal defenses of the respiratory system may suggest potential targets to prevent invasive infections of the damaged lung.

## Introduction

The airway mucosal barrier provides a critical defense against infection. These boundaries must be tightly regulated, enabling the recruitment of phagocytes when needed to fight infection due to pathogens, while simultaneously maintaining epithelial integrity through repair and regeneration. Such control is especially important in the airway of the critically ill patient, where normal clearance mechanisms are impaired by airway manipulation and sedation, aspirated opportunistic bacteria proliferate locally, penetrate across the damaged epithelial barrier and invade into the lung tissue. Insults such as ventilator induced injury, viral infection, and aspiration of acidic gastric contents allow the commensal organisms and newly acquired flora to incite clinically significant tracheitis and parenchymal lung disease. It is in this clinical setting that ventilator associated infections with organisms such as the ESKAPE pathogens (*Enterococcus faecium, Staphylococcus aureus, Klebsiella pneumoniae, Acinetobacter baumannii, Pseudomonas aeruginosa, Enterobacter* spp.) emerge to cause ongoing infection and lead to significant morbidity and mortality. Multiple factors contribute to the success of these pathogens; their genomic flexibility leading to the acquisition of novel antimicrobial resistance genes and fitness elements, ability to adapt to the metabolic constraints of the lung, and the selection of biofilm-forming isolates that express extracellular polysaccharides that elude efficient phagocytosis. Appreciating the multiple factors that underlie the pathogenesis of bacterial pneumonia, beyond the issues of antimicrobial susceptibility, will be important in developing alternative approaches toward the prevention and treatment of these common infections.

**Figure 1:**
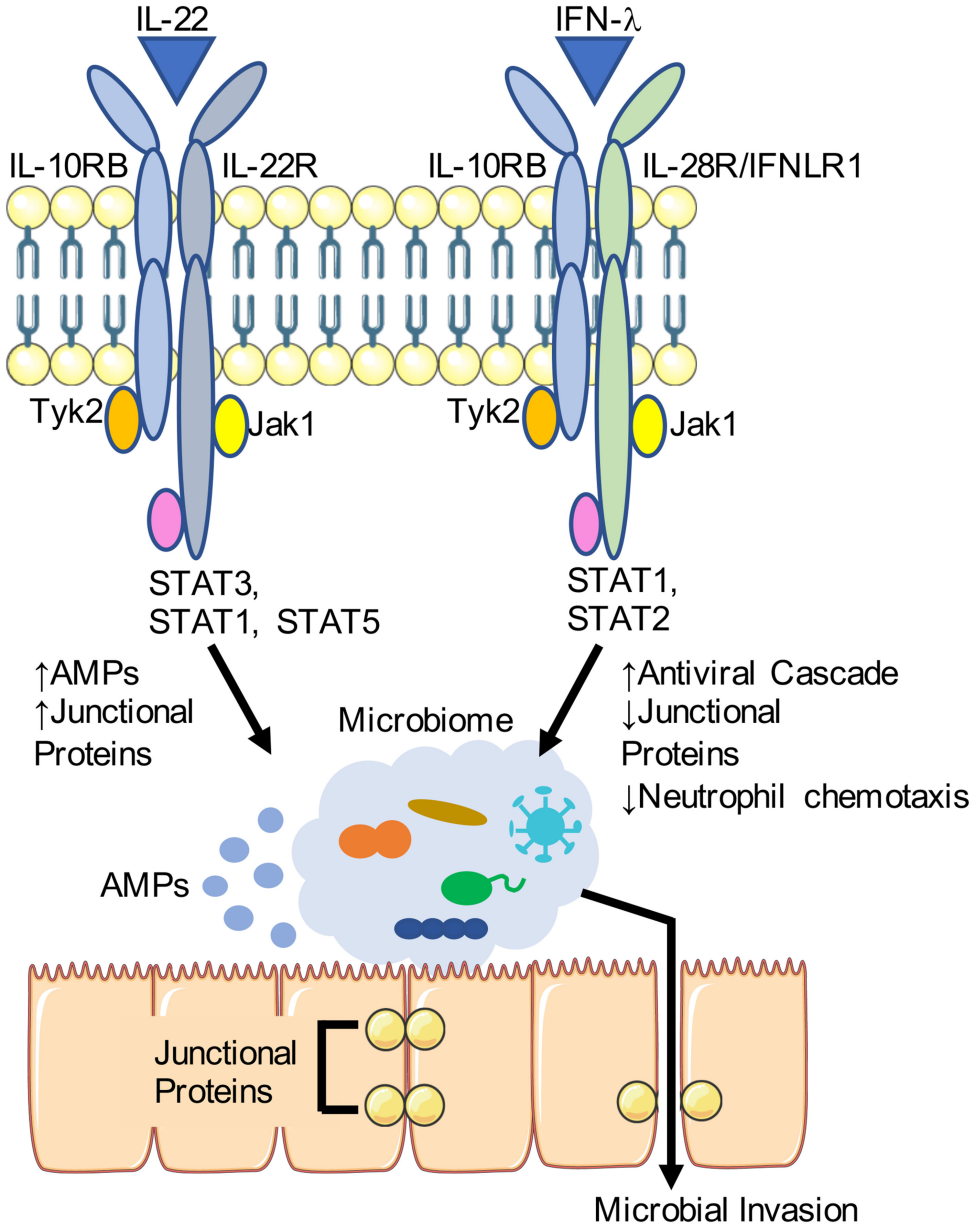
Differential effects of IL-22 and IFN-λ on the airway epithelial barrier. IL-22 and IFN-λ share a cognate receptor IL-10RB and stimulate JAK/STAT signaling. Downstream of IL-22, antimicrobial peptide (AMP) production is increased along with expression of junctional proteins. IFN-λ is induced in response to viruses such as influenza, and is associated with decreased expression of junctional protein and enhance transmigration of immune cells. Portions of this schematic was adapted by servier medical art website (smart.servier.com).

One potential approach toward preventing infection in vulnerable patient populations is through manipulation of the cytokines and interferons that normally function to protect or harm the integrity of the airway epithelial barrier. In this review we will focus upon the effects of IL-22 and interferon-λ (IFN-λ), members of the IL-10 family of cytokines which play important roles in mucosal defenses (Figure 1). Their effector receptors are generally restricted to epithelial surfaces, with some important exceptions, and these cytokines have multiple pleotropic effects that are dependent upon the inciting pathogen and the site of infection. We will focus particularly upon the participation of these two cytokines in the respiratory mucosal barrier in the setting of influenza and opportunistic bacterial infections due to the increasingly prevalent ESKAPE pathogens. Understanding how this family of cytokines influence the airway epithelial barrier in the setting of serious infection may highlight potential therapeutic targets that will enable the host to maintain this critical barrier.

## IL-22 and IFN-λ – Airway Cytokines With Shared Properties and Divergent Functions

IL-22 and IFN-λ are both important cytokines that function at mucosal barriers in response to apparent infection (Sonnenberg et al., 2011; Kotenko et al., 2019). The type III interferons (IFN-λs) are increasingly appreciated as the major interferon expressed in the context of viral infection of mucosal sites and have many structural and functional similarities with IL-22, another member of the IL-10 family of cytokines (Kotenko et al., 2019). Both IL-22 and IFN-λ signal through the class II cytokine receptors, a group of heterodimeric receptors that, when in combination, mediate responses to the IL-10 family of cytokines (Renauld, 2003). Although the specific sequence homologies within this cytokine group are limited, their protein structures are highly related and likely evolutionarily associated. The binding and activation of shared and unique receptor combinations by both IFN-λ and IL-22 lead to JAK/STAT activation and multiple responses resulting in antiviral activity, production of antimicrobial peptides, cellular proliferation and repair, and generation of acute phase reactants necessary for inflammation (Trivella et al., 2010). Both cytokines signal through the shared cognate receptor IL-10RB (Donnelly and Kotenko, 2010). IL-22 for example, binds to two copies of the IL-22R and two copies of IL-10RB with IFN-λ similarly binding to two copies of IL-28R and IL-10RB. The two cytokines appear to work synergistically as in the case of enteric viral infections such as rotavirus (Hernandez et al., 2015). Alternatively, treatment with IL-22 promotes expression of IFN-λ to inhibit replication of enteric coronavirus (Xue et al., 2017). Similarly, in *P. aeruginosa* pneumonia, production of the two proteins was additive and parallel, dampening overall pulmonary inflammation (Broquet et al., 2017, 2019). These observations suggest that in several models of infection, the related proteins IL-22 and IFN-λ work in concert.

However, there are differential kinetics of expression of these proteins dependent on the model system being studied. Dynamic expression of IFN-λ through the course of infection is observed in influenza, in which IFN-λ is produced earlier, in higher and more sustained levels in the course of infection than type 1 IFNs (Galani et al., 2017). In pneumonia caused by carbapenem-resistant ST258 *K. pneumoniae* (ST258), the kinetics of the two cytokines are distinct, with expression of IFN-λ increasing steadily with infection, coincident with the arrival of recruited monocytes, while IL-22 increases briefly at the start of infection and then again in the resolution phase (Ahn et al., 2019). In certain instances, when one signaling pathway is lost, the other is upregulated. For example, when IFN-λ signaling is absent, there is increased expression of IL-22 and antimicrobial peptides RegIIIγ and NGAL in the upper airways of mice, as well as less abundant flora (Planet et al., 2016).

The effects of these cytokines are also dependent upon the relative distribution of their receptors for these cytokines. The receptor for IL-22 (IL-22Ra1) is basally expressed in the large and small airways. In influenza and other models of infection expression is upregulated and includes the alveoli in influenza (Pociask et al., 2013; Elsegeiny et al., 2018). This dynamic regulation is an important component of the mechanism of IL-22 action and effect on the healing of the respiratory epithelium after initial lung injury. IFNLR1, the receptor for IFN-λ, is predominantly expressed in epithelial cells of the respiratory, enteric, hepatic, and integumentary systems (Galani et al., 2015; Broggi et al., 2020). Though receptors for both cytokines are generally restricted to epithelial compartments, the IFN-λ receptor is expressed in myeloid cells such as dendritic cells (Galani et al., 2015) and neutrophils (Blazek et al., 2015; Broggi et al., 2017; Galani et al., 2017). IFN-λ receptor signaling plays an important role on the function of these immune cells, discussed later in this review. Importantly, the activity of IL-22 has an additional layer of regulation with co-expression of IL-22 binding protein (IL-22BP), the soluble form of the IL-22 receptor that has neutralizing effects (Wu et al., 2008; de Moura et al., 2009; Lim et al., 2016). A similar soluble receptor for IFNRL1 has also been described but less well-studied (Witte et al., 2009). IL-22 and IFN-λ therefore have many shared characteristics and there is a growing body of evidence that signaling of these two cytokines are intimately related in regulating not only the local inflammatory environment in response to infection but also the strength of the airway epithelial barrier.

## Interleukin-22 (IL-22)

The three major roles of IL-22 in host defense are in (1) antimicrobial peptide production, (2) maintenance of epithelial barrier integrity, and (3) coordinated recruitment of immune cells in collaboration with other cytokines (Wolk et al., 2006). Production of IL-22 is regulated by pathogen recognition receptor (PRR) pathways that induce IL-23 production through toll-like receptor stimulation (TLR1/2/4/7/9) and subsequent signal transduction via MyD88, though IL-1β, TGF-β, and aryl hydrocarbon receptor also play a major role in regulation (Sonnenberg et al., 2011; Valeri and Raffatellu, 2016). IL-22 is also expressed in response to epithelial stretch in the setting of mechanical ventilation (De Winter et al., 2019). The major producers of IL-22 are T cells and innate lymphoid cells (ILCs) (Ouyang et al., 2011; Rutz et al., 2013), consistent with the cytokine's major role in immune signaling. Amongst the T cell subpopulation, T_H_17 cells appear to be the greatest producers of IL-22, with parallel production of IL-17 (Chung et al., 2006; Liang et al., 2006; Zheng et al., 2007). Other immune cell populations have been shown to produce IL-22 such as natural killer (NK), NKT, CD8^+^ T, γδ T, and dendritic cells as well as recruited monocytes (Zheng et al., 2007; Witte et al., 2010; Xu et al., 2014; Ahn et al., 2019). The targets of IL-22 are largely restricted to epithelial compartments, suggesting a critical role of this cytokine in immune cell and epithelial cell crosstalk.

The role of IL-22 however is dependent on the specific pathogen and affected host compartment, with pro-inflammatory properties in one context and reparative anti-inflammatory activities in others (Aujla and Kolls, 2009; Shabgah et al., 2017). Early studies found IL-22 upregulation in many inflammatory conditions such as rheumatoid arthritis (Roeleveld and Koenders, 2015), psoriasis (Wolk et al., 2006; Gordon et al., 2019), inflammatory bowel disease (Mizoguchi et al., 2018), asthma (Besnard et al., 2011), and hyperIgE/STAT3 mutation (Schroder, 2010), suggesting promotion of pro-inflammatory pathways. However, subsequent work has highlighted the protective function of this cytokine (Pan et al., 2004; Radaeva et al., 2004; Zenewicz et al., 2007, 2008; Sugimoto et al., 2008; Pickert et al., 2009; Barthelemy et al., 2018). At least in enteric models of viral infection, IL-22 appears to have indirect antiviral activity against rotovirus (Hernandez et al., 2015) and coronavirus (Xue et al., 2017) in concert with IFN-λ and downstream STAT1/3 signaling, though less evidence supports similar observations in the lung. IL-22 functions to upregulate the expression of junctional proteins in polarized airway epithelial cells and increase the integrity of the barrier as measured by transepithelial resistance (TEER) and permeability by dextran, bacteria, and immune cells (Ahn et al., 2019).

## IL-22 and IL-22BP Regulating the Airway Epithelial Barrier in Influenza Infection

There is substantial expression of IL-22 in the setting of viral infection, especially influenza (Ivanov et al., 2013; Kumar et al., 2013; Pociask et al., 2013; Abood et al., 2019). This has led to a number of studies establishing a role for IL-22 in maintaining epithelial barrier integrity in the context of influenza infection (Paget et al., 2012; Kumar et al., 2013; Pociask et al., 2013). Mice lacking the ability to produce IL-22 and infected with influenza virus have significant disruption of their epithelial barrier function, with significantly elevated protein in BALF (Ivanov et al., 2013). Consistent with these findings, treatment of influenza infected mice with IL-22-Fc enhanced the cell-cell junctional interactions, mediated by claudins and cadherins, and upregulated epithelial growth and proliferation demonstrating a major role of IL-22 in tissue repair and regeneration (Barthelemy et al., 2018). These mice were also found to have restoration of their barrier function as evidenced by decreased total protein and FITC-Dextran measured in the BALF and most importantly, reduced dissemination of bacteria upon pulmonary superinfection. Thus, in the context of influenza infection, IL-22 appears to be protective.

The regulation of IL-22 signaling by IL-22BP, a soluble decoy form of IL-22R that competitively inhibits IL-22 prevents downstream IL-22 signaling, is an additional layer of regulation not present in many other cytokines (Dumoutier et al., 2001; Kotenko et al., 2001; Xu et al., 2001). While IL-22 expression is increased over the course of influenza infection in mice, the amounts of IL-22BP concurrently decrease, augmenting cytokine activity in the resolution phase of infection (Ivanov et al., 2013; Abood et al., 2019; Hebert et al., 2020). In mice lacking IL-22BP and its subsequent IL-22 neutralizing activity, there was increased expression of tight junctional proteins (tjp1/2), claudin 4, occludin, and ZO-1 in airway epithelial cells isolated from these mice (Hebert et al., 2020). The associated functional impact of removing IL-22BP and therefore increased IL-22 activity, was reflected in decreased protein content in the BALF, reduced translocation of systemically administered Evans Blue dye and attenuated TEER *in vitro* (Hebert et al., 2020). The consequences of reduced IL-22BP expression in the context of influenza infection is seen with improved outcome in *Il122bp*^−/−^ mice with bacterial superinfection (Abood et al., 2019). Therefore, therapeutic strategies to titrate IL-22 expression by blocking IL-22BP may be useful clinically.

## IL-22 and Inflammation

Depending on the context, the influence of IL-22 in the response to inflammation is often due to the co-expression and regulation of other cytokines such as IL-17 and IFN-λ. Lacking receptors on immune cells, IL-22 indirectly influences the recruitment of inflammatory cells by regulating other cytokines that are co-expressed (Wolk et al., 2006, 2009; Besnard et al., 2011). In a model of bleomycin induced lung injury (Sonnenberg et al., 2010) and *K. pneumoniae* pneumonia (Aujla et al., 2008), IL-22, and IL-17A are synergistic and promote inflammation via chemokine expression and neutrophil recruitment. In the absence of IL-17A, IL-22 expression is increased and plays a protective role, preventing airway epithelial death (Sonnenberg et al., 2010). In an asthma model of inflammation, the neutralization of IL-22 in the antigen challenge phase augmented expression of IL-17 and led to increased recruitment of neutrophils and eosinophils (Besnard et al., 2011). Thus, as we will discuss below in the context of *S. aureus* infection, IL-22 plays a major role in the promotion of chemokine signaling in inflammatory processes. These studies demonstrate the variability in the role of IL-22 in inflammation that is dependent on co-expression with other inflammatory cytokines.

## *S. aureus* and IL-22

The role of IL-22 in pro-inflammatory signaling in concert with other cytokines is illustrated in the setting of *S. aureus* infection. Several virulence factors of *S. aureus* such as α-hemolysin, SEA/SEB, and proteases induce the production of IL-22 in the skin directly (Niebuhr et al., 2010; Nakatsuji et al., 2016; Orfali et al., 2018). In clinical studies, it was observed that IL-22 is elevated in the serum of patients that have skin and soft tissue infections (SSTIs) caused by *S. aureus*, along with other pro-inflammatory cytokines TNF, IFN-β, IL-17F (Alegre et al., 2016). Patients with chronic inflammation induced by atopic dermatitis, commonly associated with *S. aureus* colonization, tended to have elevated levels of IL-22, suggesting a role in this inflammatory condition (Orfali et al., 2018). Circulating peripheral blood mononuclear cells (PBMCs) in these patients also produced higher levels of IL-22 in response to staphylococcal enterotoxin A and B (SEA and SEB) *ex vivo*. Other studies indicate that IL-22 and IL-17, two highly related cytokines as noted above, are both increased and necessary for *S. aureus* control in SSTI infection (Yeaman et al., 2014; Chan et al., 2015; Marchitto et al., 2019). However, the importance of IL-22 over IL-17 is unclear as the two cytokines likely work in concert and are often produced by the same immune cell populations (Cho et al., 2010).

The induction of IL-22 expression by immune cells also plays a protective role against *S. aureus*. Several reports demonstrate a critical role of IL-22 in the defense of lung infection with *S. aureus* (Gauguet et al., 2015; Barthelemy et al., 2018; Abood et al., 2019); as shown in models using either neutralization with anti-IL-22 antibody, treatment with recombinant protein, or with transgenic mice lacking IL-22 or IL-22BP. IL-22 enhances airway barrier function, improving the outcome of *S. aureus* superinfection of the lung after influenza, as noted above. However, the beneficial effects of IL-22 expression can also be remote. Segmented filamentous bacteria (SFB) in the gut promote production of IL-22 in the BALF that was ultimately protective in *S. aureus* infection of the lung (Gauguet et al., 2015). The exact influence of IL-22 on barrier function and the colonization of SFB is difficult to delineate, as administration of IL-22-Fc lowers SFB burden (Shih et al., 2014) and SFB overgrowth is reported in the *Il22*^−/−^ mouse (McAleer et al., 2016). The beneficial effect of IL-22 has been observed in the context of nasal colonization with *S. aureus*, which increased IL-22 and subsequent expression of the AMPs, β-defensin3/4/14, S100A8/9, and Reg3γ (Mulcahy et al., 2016). In the context of damaged respiratory epithelium due to viral infection, direct mechanical ventilator injury, or aspiration of gastric contents, any disruption in the microbiome can lead to the emergence of opportunistic pathogens and bacterial superinfection or chronic colonization of the lung. The enhancement of AMP production by IL-22 plays therefore plays an important role in preventing the selection of pathogenic organisms in the compromised lung.

## *K. pneumoniae* and IL-22

IL-22 is produced in response to *Klebsiella pneumoniae*, a major cause of ventilator-associated pneumonia that results in morbidity and mortality in hospitalized patients. The major sources of IL-22 in response to *K. pneumoniae* infection are T_H_17 cells, NK cells, ILCs, and monocytes (Aujla et al., 2008; Xu et al., 2014; Ahn et al., 2019; Coorens et al., 2019). Several models have confirmed that IL-22 is critical to the clearance of *K. pneumoniae* (Aujla et al., 2008; Xu et al., 2014). In the absence of IL-22, there is significantly greater susceptibility to *K. pneumoniae*, attributable to IL-22 dependent production of lipocalin-2 (Lcn2), a potent anti-*K. pneumonaie* antimicrobial protein (Coorens et al., 2019). Several studies have confirmed the role of IL-17 in the clearance of *K. pneumoniae*, which is often co-expressed with IL-22 (Aujla et al., 2008; Chan et al., 2015; Ahn et al., 2019). As antimicrobial resistance in *K. pneumoniae* is a major problem, there is substantial interest in the role IL-22 in preventing respiratory infection with opportunistic pathogens such as *K. pneumoniae*.

*K. pneumoniae* frequently cause bacteremia and sepsis, as a complication of pneumonia. The expression of IL-22 at the airway mucosa in response, has major effects on the integrity of the epithelial barrier. In polarized airway epithelial cells infected with carbapenem resistant ST258 *K. pneumoniae* (ST258), treatment with recombinant IL-22 enhanced the barrier function *in vitro*, blocking dextran permeability as well as inhibiting neutrophil transmigration and microbial invasion (Ahn et al., 2019). However, *Il22*^−/^^−^ mice in this study, were not more susceptible to pneumonia with ST258, suggesting that other innate immune factors are also important. The behavior of ST258 in *Il22*^−/−^ mice was contrary to that seen with infection with a more virulent laboratory strain where IL-22 was critical to the host defense in a mouse model of pneumonia (Xu et al., 2014). Therefore, IL-22 has differential effects in *K. pneumoniae* infection of the lung, likely due to genetic and phenotypic differences of newer clinical isolates.

## *P. aeruginosa* and IL-22

*P. aeruginosa* is different from other opportunistic pathogens in its ability to activate an IL-22 response, though also playing a protective role of at the level of the epithelium. *P. aeruginosa* is another ESKAPE pathogen, often a cause of hospital-associated infections and an organism that readily acquires resistance to all classes of antibiotics. In cystic fibrosis (CF), where *P. aeruginosa* is a common colonizing pathogen of the lung, IL-22 is produced and is localized to the airways (Bayes et al., 2016). However, transcription levels of IL-22 in the CF lung are lower than in healthy controls which is in contrast to increased mRNA of related cytokines IL-17A and IL-23 (Aujla et al., 2008; Decraene et al., 2010). *P. aeruginosa* itself might contribute to the decreased levels of IL-22 in CF. Protease IV, part of the type 2 secretion system of *P. aeruginosa* is capable of degrading the protein directly (Guillon et al., 2017). This is a possible mechanism for a reduction in IL-22 mRNA and protein levels observed in ventilated rats that had elevated levels prior to infection with *P. aeruginosa* (De Winter et al., 2019). In addition to decreased production of IL-22, the inflamed airway of a CF patient is thought to decrease IL-22 levels through direct degradation by serine proteases produced by immune cells, namely neutrophils, in the airway (Guillon et al., 2019). This is similarly observed in chronic obstructive pulmonary disease (COPD), another model of chronic inflammation (Guillon et al., 2015). Less clear is the significance of IL-22 in the clearance of *P. aeruginosa* (Bayes et al., 2016). In the absence of IL-22, there was no difference in bacterial clearance but weight loss was attenuated suggesting a systemic inflammatory role of IL-22 in the context of *P. aeruginosa* pulmonary infection. In two reports, Broquet et al. showed that increased IL-22 led to an increase in IFN-λ in *P. aeruginosa* pneumonia, suggesting parallel expression of the two cytokines (Broquet et al., 2017, 2019). Thus, in the setting of more chronic infection such as CF and COPD, the net effect on IL-22 and IFN-λ signaling pathways may not have as clear cut effects as can be illustrated in other models of acute pneumonia.

## Therapeutic Strategies Using IL-22

The therapeutic potential of IL-22 has been studied in a variety of disease processes including GVHD of the gut, colitis, inflammatory skin disorders, and inflammatory conditions of the lung such as asthma and fibrosis (Sabat et al., 2014). Utilization of IL-22 to treat epithelial injury in the lung requires higher doses to increase transcription of its downstream effectors (SAA3, Reg3γ, S100A8/9) (Barthelemy et al., 2018) than that in the liver (SAA1/2) (Trevejo-Nunez et al., 2016; Zheng et al., 2016; Stefanich et al., 2018). However, in animal models of post-viral bacterial infection, even lower doses of IL-22 appeared to decrease bacterial burden suggesting a strong influence of off target signaling in the liver than enhance bacterial clearance elsewhere. The pharmacodynamics of systemic IL-22 administration can be established, as studies suggest that saturation of the hepatic receptors is required before agonism of receptors in the lung occurs (Barthelemy et al., 2018). At the time of this publication, a phase 2 open-label study is ongoing for the use of IL-22-Fc for IBD (ClinicalTrials.gov Identifier, NCT03650413) along with an ongoing study of the role of IL-22 in COPD (ClinicalTrials.gov Identifier, NCT02655302).

## Interferon-λ (IFN-λ)

Type III interferons (IFNs), also referred to as IFN-λ, IFNL1, IL-28A/B, or IL-29, are increasingly recognized as important contributors to mucosal defenses, with many properties entirely distinct from those of the type I IFNS, α, and β. IFN-λ is functionally an interferon but structurally related to IL-10 family (Gad et al., 2009). The IFNs as a group are primarily associated with viral infection, although it is now very clear that they contribute substantially to innate immune defenses against a wide variety of pathogens. Their role in bacterial infection is clearly illustrated by the induction of the type III interferons in response to bacterial pathogen associated molecular patterns (PAMPs). While IFN-λ signaling overlaps to some degree with the type I IFNs, there are important differences in the kinetics of expression, distribution of IFN-λ receptors, and signaling that makes the role of IFN-λ unique in the context of inflammation and infection, particularly in influenza infection.

The specifics of differential signaling between the two types of interferons are well-reviewed (Lazear et al., 2015; Kotenko et al., 2019). Induction of IFN-λ production is distinct from type I interferons, which typically requires stimulation of endosomally expressed TLRs (3/7/8/9) (Noppert et al., 2007). Both type I and III interferons are produced in response to stimulation of the cell surface receptor TLR4 (Odendall et al., 2017). Interestingly, other cell surface receptors like TLR2 also lead to dual expression, although there is relatively higher expression of IFN-λ compared to IFN-β (Odendall et al., 2017). A major distinguishing characteristic of these two classes of cytokines is that the receptors for IFNα/β are ubiquitous while those for IFN-λ are restricted to mucosal surfaces and neutrophils (Meager et al., 2005; Zhou et al., 2007; Ank et al., 2008; Maher et al., 2008; Sommereyns et al., 2008; Pulverer et al., 2010; Kroczynska et al., 2012). Unlike IL-22 and IFN-α/β that are produced by immune cells, IFN-λ is primarily made by epithelial cells and therefore thought of as an “autonomous defense system” (Kotenko et al., 2019). The influence of IFN-λ signaling is complicated, in that it is pleotropic, with competing influences on the epithelial compartment and neutrophils, a major component of the innate immune response to infection.

## *P. aeruginosa* and IFN-λ

IFN-λ is produced in response to *P. aeruginosa* infection in the lung (Cohen and Prince, 2013b; Broquet et al., 2019), playing a major role in the inflammatory response of the host. However, its role in the clearance of the bacteria is less clear with some reports suggesting a protective response and others indicating impaired bacterial clearance. In a mouse model of pneumonia, administration of exogenous IFN-λ administration led to improved lung pathology (Broquet et al., 2019). The proposed mechanism for its beneficial effects is through the reduction in neutrophils recruited to the lung and reduced IL-1β production with subsequent containment of epithelial damage (Broquet et al., 2017, 2019). This is consistent with the finding that IFN-λ receptors are expressed in neutrophils and when stimulated, chemotaxis of IL-1β-producing neutrophils, and subsequent ROS production are restricted (Blazek et al., 2015; Broggi et al., 2017; Galani et al., 2017). Moreover, the detrimental effects of IL-1β signaling in *P. aeruginosa* are well-documented (Cohen and Prince, 2013a). This is in contrast to the findings that in an *Ifnrl*^−/−^ mouse model of pneumonia, lacking the receptor for IFN-λ, clearance of *P. aeruginosa* was improved compared to a WT control (Cohen and Prince, 2013b). In the absence of IFN-λ, inflammation was attenuated by miR21 dependent reduction of programmed cell death protein 4 (PDCD4), a gene that enhances transcription of inflammatory cytokines. These conflicting findings may be the result of the differential effects of cytokine neutralization vs. blocking receptor signaling as *Ifnrl*^−/−^ tend to have higher endogenous levels of IL-22 (Planet et al., 2016). In at least the model of *P. aeruginosa*, there appears an intimate link between these two cytokines that influences the inflammatory state of the host and the function of the innate immune system.

## *K. pneumoniae* and IFN-λ

Much less data are available to assess the importance of IFN-λ on the host response to *K. pneumoniae*. IFN-λ expression and production by cultured airway epithelial cells was increased in response to ST258 *K. pneumoniae* suggesting participation in host defense (Ahn et al., 2019). IFN-λ targeted the airway epithelial barrier, increasing the permeability, and downregulating expression of junctional proteins. In a mouse model of pneumonia, IFN-λ expression peaked just as the number of recruited monocytes from the bone marrow were maximal. However, in the absence of the receptor for IFN-λ, less bacteremia, and increased clearance of ST258 *K. pneumoniae* was observed, though no difference in immune cell numbers were noted. This difference in bacterial clearance was also seen in *S. aureus* and *P. aeruginosa* (Cohen and Prince, 2013b), suggesting a unifying mechanism of pathogenesis. In the absence of IFN-λ signaling, IL-22 expression in ST258 *K. pneumoniae* pneumonia was increased with its expected effects in strengthening of the epithelial barrier and likely expression of antimicrobial peptides. Older studies show that interferons are produced in response to the capsular polysaccharide of *K. pneumoniae* (Kato et al., 1975, 1979). In fact, type II interferons and subsequent TRIF signaling is required for *K. pneumoniae* clearance (Hershman et al., 1988; van Lieshout et al., 2015). More studies need to be performed to fully understand the influence of IFN-λ in *K. pneumoniae*, particularly given the wide variability in genotype and phenotype of clinically important isolates.

## The Role of IFN-λ in *S. aureus* Superinfection

The pro-inflammatory consequences of IFN-λ production are apparent as this cytokine has a detrimental role in the setting of *S. aureus* pulmonary infection. Mice lacking the IFN-λ receptor were found to have significantly improved clearance of *S. aureus*, along with a reduction in pro-inflammatory cytokines, particularly IL-1β, as was demonstrated in a similar model of *P. aeruginosa* pneumonia (Cohen and Prince, 2013b). Similar outcomes were observed by other investigators, who suggested that IFN-λ promotes caspase-1 and neutrophil elastase activity, especially in neutrophils (Pires and Parker, 2018). Treatment of mice with excess IFN-λ delivered via an adenovirus vector similarly led to impaired *S. aureus* clearance, with no difference in AMP production, IL-17, or IL-22 levels (Rich et al., 2019). In this study, there was a major difference in the numbers of neutrophils recruited to the site of infection with impaired uptake of bacteria by neutrophils in presence of IFN-λ. Other groups have shown the influence of IFN-λ on neutrophils with impaired chemotaxis (Blazek et al., 2015; Broggi et al., 2017), inflammatory signaling (Galani et al., 2017), and reduced reactive oxygen species production (Broggi et al., 2017). Thus, the available data demonstrate that IFN-λ, in the setting of *S. aureus* infection, increases pro-inflammatory signaling, and impairs the function and/or numbers of neutrophils needed for bacterial clearance.

Understanding the influence of IFN-λ on the airway in *S. aureus* infection is important as IFN-λ is a predominating cytokine early in infection and critical to local viral containment in influenza infection (Jewell et al., 2010). *S. aureus* is a frequent bacterial superinfection post influenza in the lung, and the abundance of IFN-λ post influenza may have clinical significance. In the case of the 2009 H1N1 pandemic, the increased relative risk of mortality in children was attributed to superinfection with methicillin-resistant isolates of *S. aureus* (Randolph et al., 2011). These clinical findings are consistent with the observed increase in susceptibility of mice capable of IFN-λ signaling to MRSA nasal colonization and pneumonia post influenza as compared to mice lacking the IFN-λ receptor (Planet et al., 2016). This was attributed to relatively decreased production of the antimicrobial peptide NGAL as well as reduced expression of proteins involved in actin depolymerization. Thus, there is concern that therapeutic use of IFN-λ for influenza infection might *increase* susceptibility to *S. aureus* superinfection.

## IFN-λ and Barrier Function

As a mucosal cytokine, there are many reasons to implicate IFN-λ in the protection of the epithelial barrier. In a model of West Nile Virus encephalitis (Lazear et al., 2015), IFN-λ signaling promoted colocalization of the junctional proteins ZO-1, and Claudin-5 between endothelial cells. This mechanistically supports the conclusion that IFN-λ promotes the integrity of the blood brain barrier. IFN-λ appears to also enhance epithelial barrier function in several models of enteric infection and inflammation (Rauch et al., 2015; Odendall et al., 2017; Ferguson et al., 2019). In proteomic studies of influenza pulmonary infection in mice, IFN-λ signaling was associated with increased expression of proteins related to cytoskeletal organization, actin-binding, and depolymerization (Planet et al., 2016), although no direct consequences on pathogenesis of infection were documented. The direct influence of IFN-λ on airway epithelial cells was measured in 16HBEs treated with recombinant cytokine, with decreased expression of junctional proteins ezrin and occludin, increased dextran permeability, and increased neutrophil transmigration across a polarized monolayer of cells (Ahn et al., 2019). In contrast to the effect of IL-22, IFN-λ appears to weaken the airway epithelial barrier, though the cumulative effect on this cytokine remains unclear.

## IFN-λ, Viral Infection, and The Microbiome

The influence of IFN-λ is perhaps best characterized in the setting of influenza, with higher and more prolonged production compared to type I IFNs (Jewell et al., 2010). The diversity and abundance of the local microbiome in a murine model of influenza infection is significantly affected by IFN-λ, with enhanced selection of *S. aureus*, a major cause of bacterial superinfection, post influenza (Planet et al., 2016). Differences in the microbiome were IFN-λ dependent, as the colonizing flora in WT mice could be recapitulated by administering rIFN-λ alone to a WT mouse but not appreciated in a mouse lacking the receptor for IFN-λ. Of note, in the absence of IFN-λ signaling, production of IL-22 was increased and likely playing a major role in shaping the composition of the microbiota.

In the gut microbiome, the influence of IFN-λ is less clear. The intersection of bacterial and viral kingdoms was studied in a mouse model of norovirus (Baldridge et al., 2015). In the presence of antimicrobials, where the commensal bacterial community is reduced, IFN-λ dependent reduction of norovirus was observed. In this case, the microbial tone influenced the antiviral properties of IFN-λ. The effect of IFN-λ on the microbial community is likely important to the health of the gut epithelial barrier, but the exact mechanism by which it occurs is yet unknown.

## IFN-λ a Potential Therapy in the COVID ERA

Repurposing of immunotherapies and antivirals for the treatment of severe acute respiratory syndrome coronavirus 2 infection (SARS-CoV-2) has been a prominent approach in the treatment of coronavirus disease 2019 (COVID-19). Exploiting the natural antiviral properties of proteins such as IFN-λ has been proposed for the treatment and prevention of this disease (Prokunina-Olsson et al., 2020), particularly given the ability of IFN-λ to control viral replication and dissemination of SARS-CoV-1 (Mordstein et al., 2010) and influenza (Davidson et al., 2016; Galani et al., 2017). Many questions remain with this approach to treatment, with the unknown effects of IFN-λ on neutrophil recruitment (Blazek et al., 2015; Rich et al., 2019) and function (Broggi et al., 2017) and importantly, the propensity to develop bacterial superinfections. At the time of this submission, a proposal for a phase 2 randomized trial for subcutaneous injection of pegylated IFN-λ (IFN-λ) for the treatment of mild COVID-19 infection is underway (ClinicalTrials.gov identifier: NTC04343976). A number of safety and efficacy trials for pIFN-λ are published or in progress for the treatment of chronic hepatitis B, C, and D (Chan et al., 2015; Nelson et al., 2017; Deterding and Wedemeyer, 2019).

## Conclusions

IL-22 and IFN-λ play major roles in influencing the outcome of bacterial infections of the lung, particularly by ESKAPE pathogens and important viral pathogens such as influenza and coronavirus. Both cytokines clearly influence the integrity of the airway epithelial barrier, though likely in opposing manners, dependent upon the site of infection, the quality of the host, antecedent influenza infection, and the kinetics of infection. In the setting of the SARS-CoV-2 pandemic, in which so much pathology is attributed to the nature of the immune response (Ong et al., 2020) there is a tremendous interest in targeting pathological cytokine activity. The T_H_17 axis overall and downstream effectors Il-22 and IFN-λ are attractive targets and JAK2 inhibitors have been proposed for therapy (Wu and Yang, 2020), along with the already FDA approved drugs that target specific pro-inflammatory cytokines such tocilizumab. In general, IL-22 appears to be protective by strengthening the epithelial barrier and upregulating antimicrobial peptides. As such, it is currently in clinical trials to protect immunocompromised patients with impaired mucosal barrier function (Gao and Xiang, 2019). Conversely, as has been observed in several models, excess inflammation, especially that mediated by IL-1β (Cohen and Prince, 2013b) as might be generated by IFN-λ is detrimental to bacterial clearance. Universally, inhibiting IFN-λ signaling enhanced bacterial clearance (Cohen and Prince, 2013b; Ahn et al., 2019). However in other models of inflammation, this was balanced with the negative effects of IFN-λ on neutrophil phagocytic capacity and IL-1β production (Blazek et al., 2015; Broquet et al., 2019). As has been recognized in the COVID-19 patients, the kinetics of cytokine release is an important variable in the distinction between an effective and a pathological immune response. The therapeutic potential of IFN-λ to treat SARS-CoV2 places even greater importance on understanding the influence of this cytokine, particularly in promoting IL-22 production and its downstream beneficial effects in the lung. More studies, using pathogens isolated directly from infected patients, are required to better understand these cytokines that share the cognate receptor IL-10RB and develop therapeutic strategies to exploit their beneficial effects.

## Author Contributions

DA and AP wrote the entire manuscript and produced figures. All authors contributed to the article and approved the submitted version.

## Conflict of Interest

The authors declare that the research was conducted in the absence of any commercial or financial relationships that could be construed as a potential conflict of interest.

## References

[B1] Abood R. N. McHugh K. J. Rich H. E. Ortiz M. A. Tobin J. M. Ramanan K.. (2019). IL-22-binding protein exacerbates influenza, bacterial super-infection. Mucosal. Immunol. 12, 1231–1243. 10.1038/s41385-019-0188-7 31296910 PMC6717528

[B2] Ahn D. Wickersham M. Riquelme S. Prince A. (2019). The effects of IFN-lambda on epithelial barrier function contribute to *Klebsiella pneumoniae* ST258 pneumonia. Am. J. Respir. Cell. Mol. Biol. 60, 158–166. 10.1165/rcmb.2018-0021OC 30183325 PMC6376406

[B3] Alegre M. L. Chen L. David M. Z. Bartman C. Boyle-Vavra S. Kumar N.. (2016). Impact of *Staphylococcus aureus* USA300 colonization and skin infections on systemic immune responses in humans. J. Immunol. 197, 1118–1126. 10.4049/jimmunol.1600549 27402695 PMC4976020

[B4] Ank N. Iversen M. B. Bartholdy C. Staeheli P. Hartmann R. Jensen U. B.. (2008). An important role for type III interferon (IFN-lambda/IL-28) in TLR-induced antiviral activity. J Immunol. 180, 2474–2485. 10.4049/jimmunol.180.4.2474 18250457

[B5] Aujla S. J. Chan Y. R. Zheng M. Fei M. Askew D. J. Pociask D. A.. (2008). IL-22 mediates mucosal host defense against gram-negative bacterial pneumonia. Nat. Med. 14, 275–281. 10.1038/nm1710 18264110 PMC2901867

[B6] Aujla S. J. Kolls J. K. (2009). IL-22: a critical mediator in mucosal host defense. J. Mol. Med. 87, 451–454. 10.1007/s00109-009-0448-1 19219418

[B7] Baldridge M. T. Nice T. J. McCune B. T. Yokoyama C. C. Kambal A. Wheadon M.. (2015). Commensal microbes and interferon-lambda determine persistence of enteric murine norovirus infection. Science 347, 266–269. 10.1126/science.1258025 25431490 PMC4409937

[B8] Barthelemy A. Sencio V. Soulard D. Deruyter L. Faveeuw C. Le Goffic R.. (2018). Interleukin-22 immunotherapy during severe influenza enhances lung tissue integrity and reduces secondary bacterial systemic invasion. Infect. Immun. 86:e00706–17. 10.1128/IAI.00706-17 29661933 PMC6013680

[B9] Bayes H. K. Ritchie N. D. Ward C. Corris P. A. Brodlie M. Evans T. J. (2016). IL-22 exacerbates weight loss in a murine model of chronic pulmonary *Pseudomonas aeruginosa* infection. J. Cyst. Fibros. 15, 759–768. 10.1016/j.jcf.2016.06.008 27375092 PMC5154339

[B10] Besnard A. G. Sabat R. Dumoutier L. Renauld J. C. Willart M. Lambrecht B.. (2011). Dual role of IL-22 in allergic airway inflammation and its cross-talk with IL-17A. Am. J. Respir. Crit. Care Med. 183, 1153–1163. 10.1164/rccm.201008-1383OC 21297073

[B11] Blazek K. Eames H. L. Weiss M. Byrne A. J. Perocheau D. Pease J. E.. (2015). IFN-lambda resolves inflammation via suppression of neutrophil infiltration and IL-1beta production. J. Exp. Med. 212, 845–853. 10.1084/jem.20140995 25941255 PMC4451128

[B12] Broggi A. Granucci F. Zanoni I. (2020). Type III interferons: balancing tissue tolerance and resistance to pathogen invasion. J. Exp. Med. 217:e20190295. 10.1084/jem.20190295 31821443 PMC7037241

[B13] Broggi A. Tan Y. Granucci F. Zanoni I. (2017). IFN-lambda suppresses intestinal inflammation by non-translational regulation of neutrophil function. Nat. Immunol. 18, 1084–1093. 10.1038/ni.3821 28846084 PMC5701513

[B14] Broquet A. Besbes A. Martin J. Jacqueline C. Vourc'h M. Roquilly A.. (2019). Interleukin-22 regulates interferon lambda expression in a mice model of *pseudomonas aeruginosa* pneumonia. Mol. Immunol. 118, 52–59. 10.1016/j.molimm.2019.12.003 31855807

[B15] Broquet A. Jacqueline C. Davieau M. Besbes A. Roquilly A. Martin J.. (2017). Interleukin-22 level is negatively correlated with neutrophil recruitment in the lungs in a Pseudomonas aeruginosa pneumonia model. Sci. Rep. 7:11010. 10.1038/s41598-017-11518-0 28887540 PMC5591182

[B16] Chan L. C. Chaili S. Filler S. G. Barr K. Wang H. Kupferwasser D.. (2015). Nonredundant roles of interleukin-17A (IL-17A) and IL-22 in murine host defense against cutaneous and hematogenous infection due to methicillin-resistant *Staphylococcus aureus*. Infect. Immun. 83, 4427–4437. 10.1128/IAI.01061-15 26351278 PMC4598415

[B17] Cho J. S. Pietras E. M. Garcia N. C. Ramos R. I. Farzam D. M. Monroe H. R.. (2010). IL-17 is essential for host defense against cutaneous *Staphylococcus aureus* infection in mice. J. Clin. Invest. 120, 1762–1773. 10.1172/JCI40891 20364087 PMC2860944

[B18] Chung Y. Yang X. Chang S. H. Ma L. Tian Q. Dong C. (2006). Expression and regulation of IL-22 in the IL-17-producing CD4^+^ T lymphocytes. Cell. Res. 16, 902–907. 10.1038/sj.cr.7310106 17088898

[B19] Cohen T. S. Prince A. S. (2013a). Activation of inflammasome signaling mediates pathology of acute P. aeruginosa pneumonia. J. Clin. Invest. 123, 1630–1637. 10.1172/JCI66142 23478406 PMC3613922

[B20] Cohen T. S. Prince A. S. (2013b). Bacterial pathogens activate a common inflammatory pathway through IFNlambda regulation of PDCD4. PLoS Pathog. 9:e1003682. 10.1371/journal.ppat.1003682 24098127 PMC3789769

[B21] Coorens M. Rao A. Grafe S. K. Unelius D. Lindforss U. Agerberth B.. (2019). Innate lymphoid cell type 3-derived interleukin-22 boosts lipocalin-2 production in intestinal epithelial cells via synergy between STAT3 and NF-kappaB. J. Biol. Chem. 294, 6027–6041. 10.1074/jbc.RA118.007290 30782844 PMC6463718

[B22] Davidson S. McCabe T. M. Crotta S. Gad H. H. Hessel E. M. Beinke S.. (2016). IFNlambda is a potent anti-influenza therapeutic without the inflammatory side effects of IFNalpha treatment. EMBO Mol. Med. 8, 1099–1112. 10.15252/emmm.201606413 27520969 PMC5009813

[B23] de Moura P. R. Watanabe L. Bleicher L. Colau D. Dumoutier L. Lemaire M. M.. (2009). Crystal structure of a soluble decoy receptor IL-22BP bound to interleukin-22. FEBS Lett. 583, 1072–1077. 10.1016/j.febslet.2009.03.006 19285080

[B24] De Winter F. H. R. Jongers B. Bielen K. Mancuso D. Timbermont L. Lammens C.. (2019). Mechanical ventilation impairs IL-17 cytokine family expression in ventilator-associated pneumonia. Int. J. Mol. Sci. 20:5072. 10.3390/ijms20205072 31614857 PMC6829394

[B25] Decraene A. Willems-Widyastuti A. Kasran A. De Boeck K. Bullens D. M. Dupont L. J. (2010). Elevated expression of both mRNA and protein levels of IL-17A in sputum of stable cystic fibrosis patients. Respir. Res. 11:177. 10.1186/1465-9921-11-177 21143945 PMC3019184

[B26] Deterding K. Wedemeyer H. (2019). Beyond pegylated interferon-alpha: new treatments for hepatitis delta. AIDS Rev. 21, 126–134. 10.24875/AIDSRev.19000080 31532397

[B27] Donnelly R. P. Kotenko S. V. (2010). Interferon-lambda: a new addition to an old family. J. Interferon. Cytokine. Res. 30, 555–564. 10.1089/jir.2010.0078 20712453 PMC2925029

[B28] Dumoutier L. Lejeune D. Colau D. Renauld J. C. (2001). Cloning and characterization of IL-22 binding protein, a natural antagonist of IL-10-related T cell-derived inducible factor/IL-22. J. Immunol. 166, 7090–7095. 10.4049/jimmunol.166.12.7090 11390453

[B29] Elsegeiny W. Zheng M. Eddens T. Gallo R. L. Dai G. Trevejo-Nunez G.. (2018). Murine models of pneumocystis infection recapitulate human primary immune disorders. JCI Insight 3:e91894. 10.1172/jci.insight.91894 29925696 PMC6124425

[B30] Ferguson S. H. Foster D. M. Sherry B. Magness S. T. Nielsen D. M. Gookin J. L. (2019). Interferon-lambda3 promotes epithelial defense and barrier function against cryptosporidium parvum infection. Cell. Mol. Gastroenterol. Hepatol. 8, 1–20. 10.1016/j.jcmgh.2019.02.007 30849550 PMC6510929

[B31] Gad H. H. Dellgren C. Hamming O. J. Vends S. Paludan S. R. Hartmann R. (2009). Interferon-lambda is functionally an interferon but structurally related to the interleukin-10 family. J. Biol. Chem. 284, 20869–20875. 10.1074/jbc.M109.002923 19457860 PMC2742852

[B32] Galani I. E. Koltsida O. Andreakos E. (2015). Type III interferons (IFNs): emerging master regulators of immunity. Adv. Exp. Med. Biol. 850, 1–15. 10.1007/978-3-319-15774-0_1 26324342

[B33] Galani I. E. Triantafyllia V. Eleminiadou E. E. Koltsida O. Stavropoulos A. Manioudaki M.. (2017). Interferon-lambda mediates non-redundant front-line antiviral protection against influenza virus infection without compromising host fitness. Immunity 46, 875–890.e876. 10.1016/j.immuni.2017.04.025 28514692

[B34] Gao B. Xiang X. (2019). Interleukin-22 from bench to bedside: a promising drug for epithelial repair. Cell. Mol. Immunol. 16, 666–667. 10.1038/s41423-018-0055-6 29921965 PMC6804818

[B35] Gauguet S. D'Ortona S. Ahnger-Pier K. Duan B. Surana N. K. Lu R.. (2015). Intestinal microbiota of mice influences resistance to *Staphylococcus aureus* pneumonia. Infect. Immun. 83, 4003–4014. 10.1128/IAI.00037-15 26216419 PMC4567647

[B36] Gordon K. B. Armstrong A. W. Foley P. Song M. Shen Y. K. Li S.. (2019). Guselkumab efficacy after withdrawal is associated with suppression of serum IL-23-regulated IL-17 and IL-22 in psoriasis: VOYAGE 2 study. J. Invest. Dermatol. 139, 2437–2446.e2431. 10.1016/j.jid.2019.05.016 31207232

[B37] Guillon A. Brea D. Luczka E. Herve V. Hasanat S. Thorey C.. (2019). Inactivation of the interleukin-22 pathway in the airways of cystic fibrosis patients. Cytokine 113, 470–474. 10.1016/j.cyto.2018.10.015 30377053

[B38] Guillon A. Brea D. Morello E. Tang A. Jouan Y. Ramphal R.. (2017). *Pseudomonas aeruginosa* proteolytically alters the interleukin 22-dependent lung mucosal defense. Virulence 8, 810–820. 10.1080/21505594.2016.1253658 27792459 PMC5626239

[B39] Guillon A. Jouan Y. Brea D. Gueugnon F. Dalloneau E. Baranek T.. (2015). Neutrophil proteases alter the interleukin-22-receptor-dependent lung antimicrobial defence. Eur. Respir. J. 46, 771–782. 10.1183/09031936.00215114 26250498

[B40] Hebert K. D. McLaughlin N. Galeas-Pena M. Zhang Z. Eddens T. Govero A.. (2020). Targeting the IL-22/IL-22BP axis enhances tight junctions and reduces inflammation during influenza infection. Mucosal. Immunol. 13, 64–74. 10.1038/s41385-019-0206-9 31597930 PMC6917921

[B41] Hernandez P. P. Mahlakoiv T. Yang I. Schwierzeck V. Nguyen N. Guendel F.. (2015). Interferon-lambda and interleukin 22 act synergistically for the induction of interferon-stimulated genes and control of rotavirus infection. Nat. Immunol. 16, 698–707. 10.1038/ni.3180 26006013 PMC4589158

[B42] Hershman M. J. Polk H. C. Jr. Pietsch J. D. Kuftinec D. Sonnenfeld G. (1988). Modulation of *Klebsiella pneumoniae* infection of mice by interferon-gamma. Clin. Exp. Immunol. 72, 406–409. 3139342 PMC1541566

[B43] Ivanov S. Renneson J. Fontaine J. Barthelemy A. Paget C. Fernandez E. M.. (2013). Interleukin-22 reduces lung inflammation during influenza A virus infection and protects against secondary bacterial infection. J. Virol. 87, 6911–6924. 10.1128/JVI.02943-12 23596287 PMC3676141

[B44] Jewell N. A. Cline T. Mertz S. E. Smirnov S. V. Flano E. Schindler C.. (2010). Lambda interferon is the predominant interferon induced by influenza a virus infection *in vivo*. J. Virol. 84, 11515–11522. 10.1128/JVI.01703-09 20739515 PMC2953143

[B45] Kato N. Nakashima I. Ohta M. Naito S. Kojima T. (1979). Interferon and cytotoxic factor (cytotoxin) released in the blood of mice infected with Mycobacterium bovis BCG. *I.* enhanced production of interferon and appearance of cytotoxin stimulated by capsular polysaccharide of *Klebsiella pneumoniae* or bacterial lipopolysaccharide. Microbiol. Immunol. 23, 383–394. 10.1111/j.1348-0421.1979.tb00475.x 41163

[B46] Kato N. Nakashima I. Ota M. (1975). Interferon production in mice by the capsular polysaccharide of *Klebsiella pneumoniae*. Infect. Immun. 12, 1–6. 10.1128/IAI.12.1.1-6.1975 166921 PMC415237

[B47] Kotenko S. V. Izotova L. S. Mirochnitchenko O. V. Esterova E. Dickensheets H. Donnelly R. P.. (2001). Identification, cloning, and characterization of a novel soluble receptor that binds IL-22 and neutralizes its activity. J. Immunol. 166, 7096–7103. 10.4049/jimmunol.166.12.7096 11390454

[B48] Kotenko S. V. Rivera A. Parker D. Durbin J. E. (2019). Type III IFNs: beyond antiviral protection. Semin. Immunol. 43:101303. 10.1016/j.smim.2019.101303 31771761 PMC7141597

[B49] Kroczynska B. Sharma B. Eklund E. A. Fish E. N. Platanias L. C. (2012). Regulatory effects of programmed cell death 4 (PDCD4) protein in interferon (IFN)-stimulated gene expression and generation of type I IFN responses. Mol. Cell. Biol. 32, 2809–2822. 10.1128/MCB.00310-12 22586265 PMC3416205

[B50] Kumar P. Thakar M. S. Ouyang W. Malarkannan S. (2013). IL-22 from conventional NK cells is epithelial regenerative and inflammation protective during influenza infection. Mucosal. Immunol. 6, 69–82. 10.1038/mi.2012.49 22739232 PMC3835350

[B51] Lazear H. M. Nice T. J. Diamond M. S. (2015). Interferon-lambda: immune functions at barrier surfaces and beyond. Immunity 43, 15–28. 10.1016/j.immuni.2015.07.001 26200010 PMC4527169

[B52] Liang S. C. Tan X. Y. Luxenberg D. P. Karim R. Dunussi-Joannopoulos K. Collins M.. (2006). Interleukin (IL)-22 and IL-17 are coexpressed by Th17 cells and cooperatively enhance expression of antimicrobial peptides. J. Exp. Med. 203, 2271–2279. 10.1084/jem.20061308 16982811 PMC2118116

[B53] Lim C. Hong M. Savan R. (2016). Human IL-22 binding protein isoforms act as a rheostat for IL-22 signaling. Sci. Signal. 9:ra95. 10.1126/scisignal.aad9887 27678220

[B54] Maher S. G. Sheikh F. Scarzello A. J. Romero-Weaver A. L. Baker D. P. Donnelly R. P.. (2008). IFNalpha and IFNlambda differ in their antiproliferative effects and duration of JAK/STAT signaling activity. Cancer Biol. Ther. 7, 1109–1115. 10.4161/cbt.7.7.6192 18698163 PMC2435218

[B55] Marchitto M. C. Dillen C. A. Liu H. Miller R. J. Archer N. K. Ortines R. V.. (2019). Clonal Vgamma6(+)Vdelta4(+) T cells promote IL-17-mediated immunity against *Staphylococcus aureus* skin infection. Proc. Natl. Acad. Sci. U.S.A. 116, 10917–10926. 10.1073/pnas.1818256116 31088972 PMC6561199

[B56] McAleer J. P. Nguyen N. L. Chen K. Kumar P. Ricks D. M. Binnie M.. (2016). Pulmonary Th17 antifungal immunity is regulated by the gut microbiome. J. Immunol. 197, 97–107. 10.4049/jimmunol.1502566 27217583 PMC4912941

[B57] Meager A. Visvalingam K. Dilger P. Bryan D. Wadhwa M. (2005). Biological activity of interleukins-28 and−29: comparison with type I interferons. Cytokine 31, 109–118. 10.1016/j.cyto.2005.04.003 15899585

[B58] Mizoguchi A. Yano A. Himuro H. Ezaki Y. Sadanaga T. Mizoguchi E. (2018). Clinical importance of IL-22 cascade in IBD. J. Gastroenterol. 53, 465–474. 10.1007/s00535-017-1401-7 29075900 PMC5866830

[B59] Mordstein M. Neugebauer E. Ditt V. Jessen B. Rieger T. Falcone V.. (2010). Lambda interferon renders epithelial cells of the respiratory and gastrointestinal tracts resistant to viral infections. J. Virol. 84, 5670–5677. 10.1128/JVI.00272-10 20335250 PMC2876583

[B60] Mulcahy M. E. Leech J. M. Renauld J. C. Mills K. H. McLoughlin R. M. (2016). Interleukin-22 regulates antimicrobial peptide expression and keratinocyte differentiation to control *Staphylococcus aureus* colonization of the nasal mucosa. Mucosal. Immunol. 9, 1429–1441. 10.1038/mi.2016.24 27007677

[B61] Nakatsuji T. Chen T. H. Two A. M. Chun K. A. Narala S. Geha R. S.. (2016). *Staphylococcus aureus* exploits epidermal barrier defects in atopic dermatitis to trigger cytokine expression. J. Invest. Dermatol. 136, 2192–2200. 10.1016/j.jid.2016.05.127 27381887 PMC5103312

[B62] Nelson M. Rubio R. Lazzarin A. Romanova S. Luetkemeyer A. Conway B.. (2017). Safety and efficacy of pegylated interferon lambda, ribavirin, and daclatasvir in HCV and HIV-coinfected patients. J. Interferon. Cytokine. Res. 37, 103–111. 10.1089/jir.2016.0082 28282271

[B63] Niebuhr M. Scharonow H. Gathmann M. Mamerow D. Werfel T. (2010). Staphylococcal exotoxins are strong inducers of IL-22: a potential role in atopic dermatitis. J. Allergy Clin. Immunol. 126, 1176–1183.e1174. 10.1016/j.jaci.2010.07.041 20864149

[B64] Noppert S. J. Fitzgerald K. A. Hertzog P. J. (2007). The role of type I interferons in TLR responses. Immunol. Cell. Biol. 85, 446–457. 10.1038/sj.icb.7100099 17667935

[B65] Odendall C. Voak A. A. Kagan J. C. (2017). Type III IFNs are commonly induced by bacteria-sensing TLRs and reinforce epithelial barriers during infection. J. Immunol. 199, 3270–3279. 10.4049/jimmunol.1700250 28954888 PMC5679450

[B66] Ong E. Z. Chan Y. F. Z. Leong W. Y. Lee N. M. Y. Kalimuddin S. Haja Mohideen S. M. (2020). A dynamic immune response shapes COVID-19 progression. Cell Host Microbe. S1931–3128, 30185–2. 10.1016/j.chom.2020.03.021 PMC7192089 32359396

[B67] Orfali R. L. da Silva Oliveira L. M. de Lima J. F. de Carvalho G. C. Ramos Y. A. L. Pereira N. Z.. (2018). *Staphylococcus aureus* enterotoxins modulate IL-22-secreting cells in adults with atopic dermatitis. Sci. Rep. 8:6665. 10.1038/s41598-018-25125-0 29703987 PMC5923268

[B68] Ouyang W. Rutz S. Crellin N. K. Valdez P. A. Hymowitz S. G. (2011). Regulation and functions of the IL-10 family of cytokines in inflammation and disease. Annu. Rev. Immunol. 29, 71–109. 10.1146/annurev-immunol-031210-101312 21166540

[B69] Paget C. Ivanov S. Fontaine J. Renneson J. Blanc F. Pichavant M.. (2012). Interleukin-22 is produced by invariant natural killer T lymphocytes during influenza A virus infection: potential role in protection against lung epithelial damages. J. Biol. Chem. 287, 8816–8829. 10.1074/jbc.M111.304758 22294696 PMC3308738

[B70] Pan H. Hong F. Radaeva S. Gao B. (2004). Hydrodynamic gene delivery of interleukin-22 protects the mouse liver from concanavalin A-, carbon tetrachloride-, and Fas ligand-induced injury via activation of STAT3. Cell. Mol. Immunol. 1, 43–49. 16212920

[B71] Pickert G. Neufert C. Leppkes M. Zheng Y. Wittkopf N. Warntjen M.. (2009). STAT3 links IL-22 signaling in intestinal epithelial cells to mucosal wound healing. J. Exp. Med. 206, 1465–1472. 10.1084/jem.20082683 19564350 PMC2715097

[B72] Pires S. Parker D. (2018). IL-1beta activation in response to *Staphylococcus aureus* lung infection requires inflammasome-dependent and independent mechanisms. Eur. J. Immunol. 48, 1707–1716. 10.1002/eji.201847556 30051912 PMC6394835

[B73] Planet P. J. Parker D. Cohen T. S. Smith H. Leon J. D. Ryan C.. (2016). Lambda interferon restructures the nasal microbiome and increases susceptibility to *Staphylococcus aureus* superinfection. mBio 7, e01939–e01915. 10.1128/mBio.01939-15 26861017 PMC4752601

[B74] Pociask D. A. Scheller E. V. Mandalapu S. McHugh K. J. Enelow R. I. Fattman C. L.. (2013). IL-22 is essential for lung epithelial repair following influenza infection. Am. J. Pathol. 182, 1286–1296. 10.1016/j.ajpath.2012.12.007 23490254 PMC3620404

[B75] Prokunina-Olsson L. Alphonse N. Dickenson R. E. Durbin J. E. Glenn J. S. Hartmann R.. (2020). COVID-19 and emerging viral infections: the case for interferon lambda. J. Exp. Med. 217:e20200653. 10.1084/jem.20200653 32289152 PMC7155807

[B76] Pulverer J. E. Rand U. Lienenklaus S. Kugel D. Zietara N. Kochs G.. (2010). Temporal and spatial resolution of type I and III interferon responses *in vivo*. J. Virol. 84, 8626–8638. 10.1128/JVI.00303-10 20573823 PMC2919002

[B77] Radaeva S. Sun R. Pan H. N. Hong F. Gao B. (2004). Interleukin 22 (IL-22) plays a protective role in T cell-mediated murine hepatitis: IL-22 is a survival factor for hepatocytes via STAT3 activation. Hepatology 39, 1332–1342. 10.1002/hep.20184 15122762

[B78] Randolph A. G. Vaughn F. Sullivan R. Rubinson L. Thompson B. T. Yoon G.. (2011). Critically ill children during the 2009-2010 influenza pandemic in the United States. Pediatrics 128, e1450–1458. 10.1542/peds.2011-0774 22065262 PMC3387899

[B79] Rauch I. Rosebrock F. Hainzl E. Heider S. Majoros A. Wienerroither S.. (2015). Noncanonical effects of IRF9 in intestinal inflammation: more than type I and type III interferons. Mol. Cell. Biol. 35, 2332–2343. 10.1128/MCB.01498-14 25918247 PMC4456449

[B80] Renauld J. C. (2003). Class II cytokine receptors and their ligands: key antiviral and inflammatory modulators. Nat. Rev. Immunol. 3, 667–676. 10.1038/nri1153 12974481

[B81] Rich H. E. McCourt C. C. Zheng W. Q. McHugh K. J. Robinson K. M. Wang J.. (2019). Interferon lambda inhibits bacterial uptake during influenza superinfection. Infect. Immun. 87:e00114–19. 10.1128/IAI.00114-19 30804099 PMC6479047

[B82] Roeleveld D. M. Koenders M. I. (2015). The role of the Th17 cytokines IL-17 and IL-22 in rheumatoid arthritis pathogenesis and developments in cytokine immunotherapy. Cytokine 74, 101–107. 10.1016/j.cyto.2014.10.006 25466295

[B83] Rutz S. Eidenschenk C. Ouyang W. (2013). IL-22, not simply a Th17 cytokine. Immunol. Rev. 252, 116–132. 10.1111/imr.12027 23405899

[B84] Sabat R. Ouyang W. Wolk K. (2014). Therapeutic opportunities of the IL-22-IL-22R1 system. Nat. Rev. Drug Discov. 13, 21–38. 10.1038/nrd4176 24378801

[B85] Schroder J. M. (2010). The role of keratinocytes in defense against infection. Curr. Opin. Infect. Dis. 23, 106–110. 10.1097/QCO.0b013e328335b004 20010101

[B86] Shabgah A. G. Navashenaq J. G. Shabgah O. G. Mohammadi H. Sahebkar A. (2017). Interleukin-22 in human inflammatory diseases and viral infections. Autoimmun. Rev. 16, 1209–1218. 10.1016/j.autrev.2017.10.004 29037907

[B87] Shih V. F. Cox J. Kljavin N. M. Dengler H. S. Reichelt M. Kumar P.. (2014). Homeostatic IL-23 receptor signaling limits Th17 response through IL-22-mediated containment of commensal microbiota. Proc. Natl. Acad. Sci. U.S.A. 111, 13942–13947. 10.1073/pnas.1323852111 25201978 PMC4183330

[B88] Sommereyns C. Paul S. Staeheli P. Michiels T. (2008). IFN-lambda (IFN-lambda) is expressed in a tissue-dependent fashion and primarily acts on epithelial cells *in vivo*. PLoS Pathog. 4:e1000017. 10.1371/journal.ppat.1000017 18369468 PMC2265414

[B89] Sonnenberg G. F. Fouser L. A. Artis D. (2011). Border patrol: regulation of immunity, inflammation and tissue homeostasis at barrier surfaces by IL-22. Nat. Immunol. 12, 383–390. 10.1038/ni.2025 21502992

[B90] Sonnenberg G. F. Nair M. G. Kirn T. J. Zaph C. Fouser L. A. Artis D. (2010). Pathological versus protective functions of IL-22 in airway inflammation are regulated by IL-17A. J. Exp. Med. 207, 1293–1305. 10.1084/jem.20092054 20498020 PMC2882840

[B91] Stefanich E. G. Rae J. Sukumaran S. Lutman J. Lekkerkerker A. Ouyang W. (2018). Pre-clinical and translational pharmacology of a human interleukin-22 IgG fusion protein for potential treatment of infectious or inflammatory diseases. Biochem. Pharmacol. 152, 224–235. 10.1016/j.bcp.2018.03.031 29608910

[B92] Sugimoto K. Ogawa A. Mizoguchi E. Shimomura Y. Andoh A. Bhan A. K.. (2008). IL-22 ameliorates intestinal inflammation in a mouse model of ulcerative colitis. J. Clin. Invest. 118, 534–544. 10.1172/JCI33194 18172556 PMC2157567

[B93] Trevejo-Nunez G. Elsegeiny W. Conboy P. Chen K. Kolls J. K. (2016). Critical role of IL-22/IL22-RA1 signaling in pneumococcal pneumonia. J. Immunol. 197, 1877–1883. 10.4049/jimmunol.1600528 27456484 PMC4992592

[B94] Trivella D. B. Ferreira-Junior J. R. Dumoutier L. Renauld J. C. Polikarpov I. (2010). Structure and function of interleukin-22 and other members of the interleukin-10 family. Cell. Mol. Life Sci. 67, 2909–2935. 10.1007/s00018-010-0380-0 20454917 PMC11115847

[B95] Valeri M. Raffatellu M. (2016). Cytokines IL-17 and IL-22 in the host response to infection. Pathog. Dis. 74:ftw111. 10.1093/femspd/ftw111 27915228 PMC5975231

[B96] van Lieshout M. H. Florquin S. Van't Veer C. de Vos A. F. van der Poll T. (2015). TIR-Domain-containing adaptor-inducing interferon-beta (TRIF) mediates antibacterial defense during gram-negative pneumonia by inducing interferon-x03B3. J. Innate. Immun. 7, 637–646. 10.1159/000430913 26065469 PMC6738753

[B97] Witte E. Witte K. Warszawska K. Sabat R. Wolk K. (2010). Interleukin-22: a cytokine produced by T, NK and NKT cell subsets, with importance in the innate immune defense and tissue protection. Cytokine. Growth Factor. Rev. 21, 365–379. 10.1016/j.cytogfr.2010.08.002 20870448

[B98] Witte K. Gruetz G. Volk H. D. Looman A. C. Asadullah K. Sterry W.. (2009). Despite IFN-lambda receptor expression, blood immune cells, but not keratinocytes or melanocytes, have an impaired response to type III interferons: implications for therapeutic applications of these cytokines. Genes. Immun. 10, 702–714. 10.1038/gene.2009.72 19798076

[B99] Wolk K. Haugen H. S. Xu W. Witte E. Waggie K. Anderson M.. (2009). IL-22 and IL-20 are key mediators of the epidermal alterations in psoriasis while IL-17 and IFN-gamma are not. J. Mol. Med. 87, 523–536. 10.1007/s00109-009-0457-0 19330474

[B100] Wolk K. Witte E. Wallace E. Docke W. D. Kunz S. Asadullah K.. (2006). IL-22 regulates the expression of genes responsible for antimicrobial defense, cellular differentiation, and mobility in keratinocytes: a potential role in psoriasis. Eur. J. Immunol. 36, 1309–1323. 10.1002/eji.200535503 16619290

[B101] Wu D. Yang X. O. (2020). TH17 responses in cytokine storm of COVID-19: an emerging target of JAK2 inhibitor fedratinib. J. Microbiol. Immunol. Infect, 53:368–70. 10.1016/j.jmii.2020.03.005 32205092 PMC7156211

[B102] Wu P. W. Li J. Kodangattil S. R. Luxenberg D. P. Bennett F. Martino M.. (2008). IL-22R, IL-10R2, and IL-22BP binding sites are topologically juxtaposed on adjacent and overlapping surfaces of IL-22. J. Mol. Biol. 382, 1168–1183. 10.1016/j.jmb.2008.07.046 18675824

[B103] Xu W. Presnell S. R. Parrish-Novak J. Kindsvogel W. Jaspers S. Chen Z.. (2001). A soluble class II cytokine receptor, IL-22RA2, is a naturally occurring IL-22 antagonist. Proc. Natl. Acad. Sci. U.S.A. 98, 9511–9516. 10.1073/pnas.171303198 11481447 PMC55483

[B104] Xu X. Weiss I. D. Zhang H. H. Singh S. P. Wynn T. A. Wilson M. S.. (2014). Conventional NK cells can produce IL-22 and promote host defense in *Klebsiella pneumoniae* pneumonia. J. Immunol. 192, 1778–1786. 10.4049/jimmunol.1300039 24442439 PMC3995347

[B105] Xue M. Zhao J. Ying L. Fu F. Li L. Ma Y.. (2017). IL-22 suppresses the infection of porcine enteric coronaviruses and rotavirus by activating STAT3 signal pathway. Antiviral. Res. 142, 68–75. 10.1016/j.antiviral.2017.03.006 28322925 PMC7113769

[B106] Yeaman M. R. Filler S. G. Chaili S. Barr K. Wang H. Kupferwasser D.. (2014). Mechanisms of NDV-3 vaccine efficacy in MRSA skin versus invasive infection. Proc. Natl. Acad. Sci. U.S.A. 111, E5555–5563. 10.1073/pnas.1415610111 25489065 PMC4280579

[B107] Zenewicz L. A. Yancopoulos G. D. Valenzuela D. M. Murphy A. J. Karow M. Flavell R. A. (2007). Interleukin-22 but not interleukin-17 provides protection to hepatocytes during acute liver inflammation. Immunity 27, 647–659. 10.1016/j.immuni.2007.07.023 17919941 PMC2149911

[B108] Zenewicz L. A. Yancopoulos G. D. Valenzuela D. M. Murphy A. J. Stevens S. Flavell R. A. (2008). Innate and adaptive interleukin-22 protects mice from inflammatory bowel disease. Immunity 29, 947–957. 10.1016/j.immuni.2008.11.003 19100701 PMC3269819

[B109] Zheng M. Horne W. McAleer J. P. Pociask D. Eddens T. Good M.. (2016). Therapeutic role of interleukin 22 in experimental intra-abdominal *Klebsiella pneumoniae* infection in mice. Infect. Immun. 84, 782–789. 10.1128/IAI.01268-15 26729763 PMC4771339

[B110] Zheng Y. Danilenko D. M. Valdez P. Kasman I. Eastham-Anderson J. Wu J.. (2007). Interleukin-22, a T(H)17 cytokine, mediates IL-23-induced dermal inflammation and acanthosis. Nature 445, 648–651. 10.1038/nature05505 17187052

[B111] Zhou Z. Hamming O. J. Ank N. Paludan S. R. Nielsen A. L. Hartmann R. (2007). Type III interferon (IFN) induces a type I IFN-like response in a restricted subset of cells through signaling pathways involving both the Jak-STAT pathway and the mitogen-activated protein kinases. J. Virol. 81, 7749–7758. 10.1128/JVI.02438-06 17507495 PMC1933366

